# Structural basis for Rab23 activation and a loss-of-function mutation in Carpenter syndrome

**DOI:** 10.1016/j.jbc.2024.108036

**Published:** 2024-11-29

**Authors:** Yat Yin Chau, Hanbin Liang, Wai Lam Tung, Catherine Hong Huan Hor, Wei Shen Aik

**Affiliations:** Department of Chemistry, Hong Kong Baptist University, Kowloon Tong, Hong Kong, China

**Keywords:** Carpenter Syndrome, genetic disease, small GTPase, rab, development, X-ray crystallography

## Abstract

Rab23 is a member of the Rab family of small GTPases. It plays crucial roles in Hedgehog signaling, ciliary transport, and embryonic development. As a small GTPase, Rab23 cycles between the GDP-bound inactivated state and the GTP-bound activated state. Mutations in Rab23 are directly implicated in Carpenter syndrome, a development disorder characterized by deformed skulls, abnormal fingers or toes, and intellectual disabilities. Several clinical point mutations, *for example*, M12K, C85R, and Y79del, have been found to occur within the GTPase domain. However, the mechanisms of activation of Rab23 and pathogenesis of its clinical mutants are still unclear with limited structural information. So far, there are only two reported crystal structures of mouse Rab23 in complex with GDP. Here, we determined high-resolution crystal structures of human Rab23 and the human Rab23 Y79del clinical mutant, in complex with GDP and GMPPNP, a nonhydrolysable GTP analog, respectively. Supported by *in vitro* biochemical and functional analyses, we demonstrated that the Y79 deletion mutant exhibited structural distortions in the switch II region relative to that of the WT. The structural changes potentially disrupted the binding of Rab23 Y79del to its interacting partners, thus leading to a loss-of-function and the development of Carpenter syndrome.

Rab23 is a small GTPase from the Rab family that plays important roles in vesicle transport and membrane trafficking (1). It also negatively regulates the Sonic Hedgehog (Shh) pathway (2, 3), which controls cell and tissue proliferation, polarization, and differentiation that are vital in embryonic development (4, 5, 6, 7, 8). In humans, mutations in the Rab23 gene are directly implicated in Carpenter syndrome (9, 10), a developmental disorder associated with developmental abnormalities such as cranial deformity, extra and short fingers or toes (11, 12). Additionally, intellectual disabilities, obesity, and heart diseases are also presented in some Carpenter syndrome patients (13, 14). Carpenter syndrome–associated mutations in the Rab23 gene reported to date include missense point mutations, in-frame deletions, and truncating mutations (9, 10). Three of the point mutations, M12K, C85R, and Y79del (Tyr79 deletion), are located in the GTPase domain of Rab23 (Fig. 1) (10). One recent *in vitro* studies showed abnormal GTP turnover and reduced ciliation in the M12K and C85R mutants (15). However, the functional consequences caused by the Y79del mutation have not yet been carefully studied.

**Figure 1:**
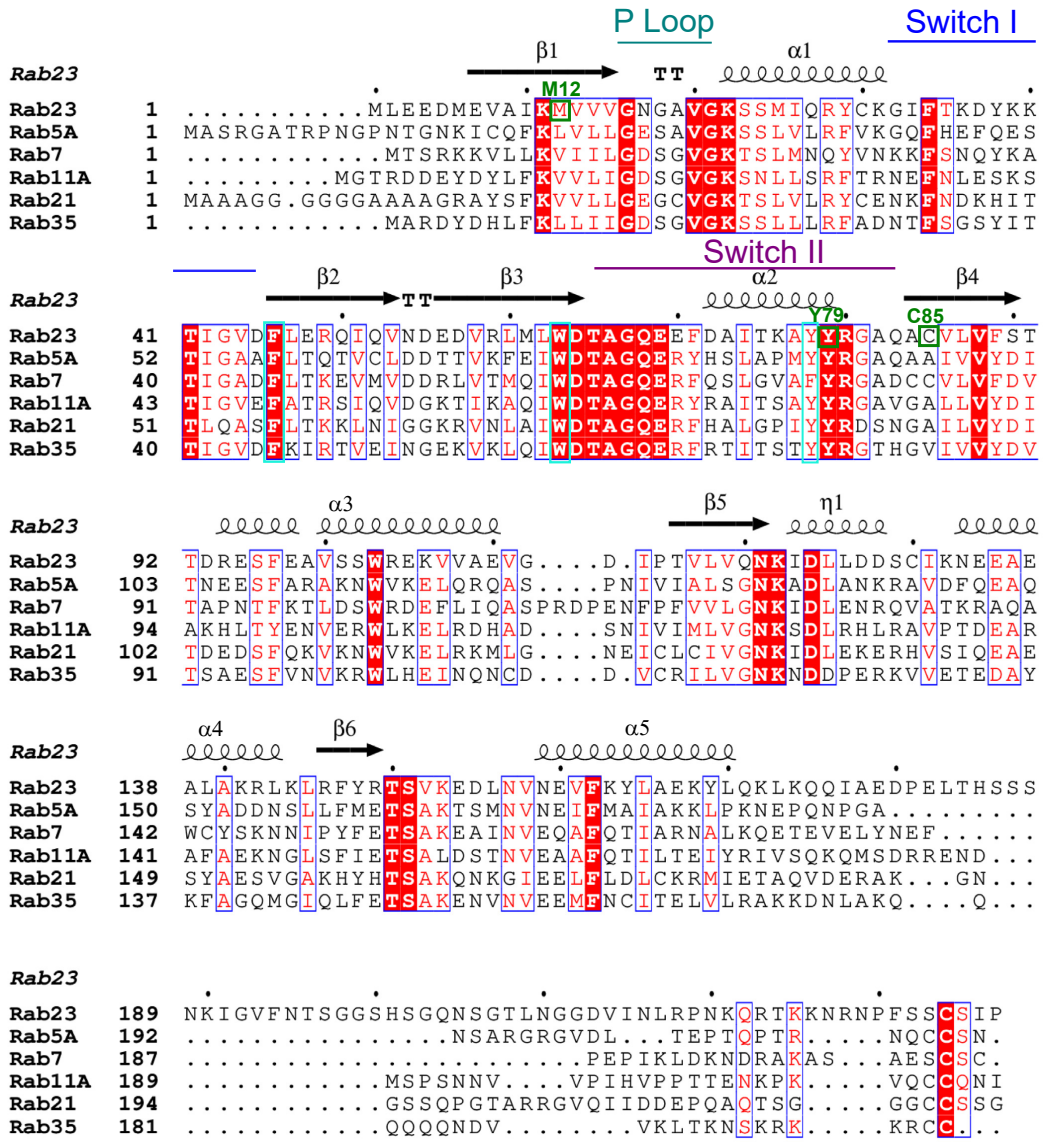
**Multiple sequence alignment of human Rab23 with representatives from the human Rab GTPase family (Rab5A, Rab7, Rab11A, Rab21, and Rab35).** Secondary structure is based on the crystal structure of the human Rab23-GDP complex (PDB ID 8YL3). Strict identity residues are indicated by *white* characters in *redboxes*; similar residues are indicated by *red* characters and *whiteboxes*; *blue* frames indicate similarity across groups. M12, C85, and Y79 of Rab23 that are mutated in Carpenter Syndrome are indicated in *green boxes* and fonts. Conserved hydrophobic triads are indicated in *cyanboxes*. Secondary structure symbols: α, α-helix; η, 3_10_-helix; β, β-strand; TT, tight turn. The sequence alignment was performed using Clustal Omega (48) and the alignment figure was produced using Espript 3.0 (https://espript.ibcp.fr) (49).

As a member of the Rab GTPase family (Fig. 1), Rab23 acts as a molecular switch by cycling between the GDP-bound inactivated (OFF) state and the GTP-bound activated (ON) state. The exchange of GDP to GTP in Rab and Ras proteins is aided by their respective guanine exchange factors (GEFs) (16). All members of the Rab GTPase family contain a conserved small GTPase fold with six β-strands and five α-helices (Fig. 1). The switch I (in between α1-helix and β2-strand) and switch II (in between strands β3 and β4) regions (Fig. 1) of small GTPases adopt different conformations depending on its activation states (17). When switched ON, Rab proteins can recruit effectors, such as motor proteins, tethering factors, and sorting adaptor proteins for downstream processes (17, 18). To switch Rab proteins off, a GTPase-activating protein (GAP) will be needed to stimulate the hydrolysis of GTP to GDP and return the Rab proteins to the inactivated state. Overexpression of the ecotropic viral integration site 5-like protein (EVI5L) has been shown to increase the GTPase activity of Rab23, suggesting that EVI5L is a Rab23-specific GAP (19). Biochemical/co-IP studies have identified the Inturned-Fuzzy (Intu-Fuz) complex as GEF of Rab23 (15, 20) and a ciliary protein, kinesin family member 17 (KIF17), as a potential Rab23 effector (21, 22). Despite the identification of these Rab23 interactors, biochemical and structural characterizations of Rab23 are still lacking. To date, only two crystal structures of mouse Rab23 (mRab23) in complex with GDP had been determined (23) but human Rab23 (hRab23), either the active or inactive form, has not been structurally characterized. The lack of structural information of hRab23 activation has also limited our understanding of how hRab23 mutations lead to the development of Carpenter syndrome.

Here, we report high-resolution crystal structures of the GTPase domain (residues 7–172) of hRab23 (hRab23_7-172_) in complex with GDP and guanosine 5′-(β-γ-imido)-triphosphate (GMPPNP), a stable GTP analog, respectively, to elucidate the structural conformations of the OFF and ON states of hRab23. To obtain structural insights into the pathogenesis of Carpenter syndrome implicating mutated Rab23, we determined crystal structures of a clinically relevant hRab23 mutant carrying a Tyr79 deletion (Y79del), in complex with GDP and GMPPNP, respectively. We also performed *in vitro* biochemical and cellular functional studies to support our structural findings. These combined results provided structural insights into the activation of hRab23 as well as its loss-of-function resulting from the Y79del point mutation.

## Results

### Overall structure of hRab23

**Figure 2:**
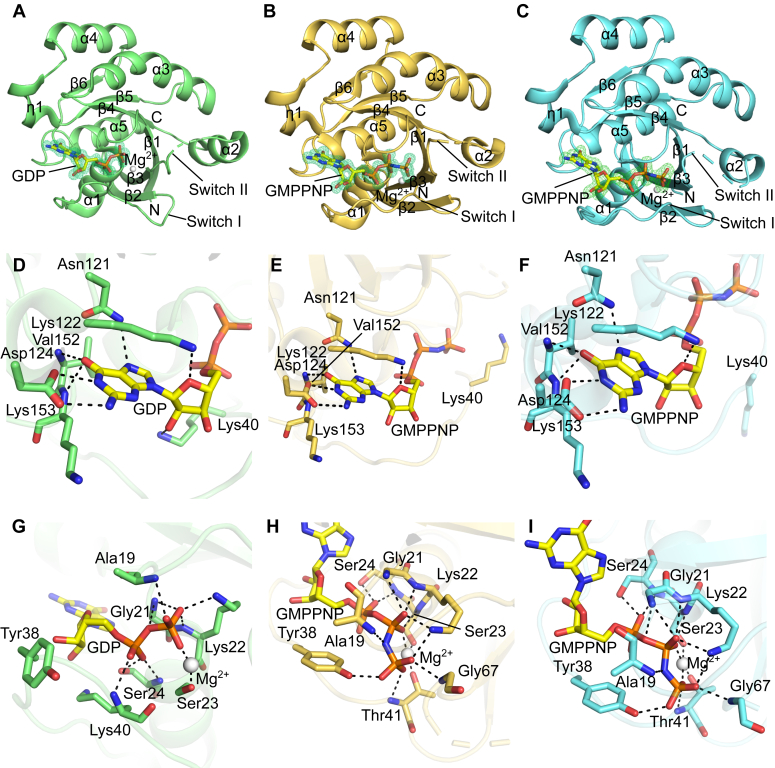
**Crystal structures of hRab23**_**7-172**_**in complex with GDP and GMPPNP.** Overall views of the structures of (*A*) the hRab23_7-172_–GDP complex (resolution 1.20 Å) (PDB ID 8YL3), (*B*) the hRab23_7-172_–GMPPNP complex (resolution 1.35 Å) (PDB ID 8YIM), and (*C*) the hRab23_7-172_–GMPPNP complex (resolution 1.80 Å) (PDB ID 8YNR). Views of the nucleotide-binding sites of (*D*) the hRab23_7-172_–GDP complex (PDB ID 8YL3), (*E*) the hRab23_7-172_–GMPPNP complex (space group *P*3_1_ 2 1) (PDB ID 8YIM), and (*F*) the hRab23_7-172_–GMPPNP complex (space group *P*2 2_1_ 2_1_) (PDB ID 8YNR). Views of the phosphate-binding sites of (*G*) the hRab23_7-172_–GDP complex (PDB ID 8YL3), (*H*) the hRab23_7-172_–GMPPNP complex (space group *P*3_1_ 2 1) (PDB ID 8YIM), and (*I*) the hRab23_7-172_–GMPPNP complex (space group *P*2 2_1_ 2_1_) (PDB ID 8YNR). GDP/GMPPNP are shown in sticks (C, *yellow*; O, *red*; N, *blue*; P, *orange*); *green* mesh represents the mF_o_-DF_c_ OMIT map of the GDP/GMPPNP ligand (contour = 3*σ*); Mg^2+^ ions are shown in *white* spheres; hydrogen bond or electrostatic interactions are indicated by *black dashes*.

To elucidate the conformational changes of hRab23 when switching between the ON and OFF state, we determined, by X-ray crystallography, a structure of hRab23_7-172_ (residues 7–172) in complex with GDP (Fig. 2*A*), representing the OFF state, and two structures of hRab23_7-172_ in complex with GMPPNP, representing the ON state. The hRab23_7-172_-GDP structure (resolution 1.20 Å) (PDB ID 8YL3) was solved by molecular replacement using an mRab23-GDP structure (PDB ID 1Z2A) as a search model. The hRab23_7-172_-GMPPNP structures (Fig. 2, *B* and *C*) were derived from crystals of different conditions and space groups. The hRab23_7-172_-GMPPNP structure with space group *P*3_1_ 2 1 was determined to a resolution of 1.35 Å (PDB ID 8YIM) while the other hRab23_7-172_-GMPPNP structure with space group *P*2 2_1_ 2_1_ was determined to a resolution of 1.80 Å (PDB ID 8YNR). Both structures were also solved by molecular replacement.

The structures of the hRab23 in complex with GDP or GMPPNP revealed a typical small GTPase fold of the Ras and Rab family proteins (Fig. 1), containing six β-strands and five α-helices (24) (Fig. 2, *A*–*C*). All the six β-strands form a single β-sheet, which is sandwiched by the α-helices and a short 3_10_ helix on both sides of the sheet. Five of the β-strands are parallel and one of them, strand β2, is anti-parallel to the rest. The switch I region (between α1 and β2) and the Interswitch region (a β-sheet containing the β2 and β3 strands) are well ordered in all three hRab23_7-172_ structures. The switch II region, which features a loop and an α-helix (α2) between the β3 and β4 strands, is partially ordered. The conformations of the switch I and II regions confer an activated (ON) or inactivated (OFF) state of small GTPases. A GDP or GMPPNP ligand and the cofactor Mg^2+^ can be observed in the nucleotide-binding pocket of hRab23_7-172_ (Fig. 2, *A*–*C*). The GDP/GMPPNP nucleotide is enclosed by four loop regions (loops between β1-α1 (P-loop) (25), α1-β2 (Switch I), β5-α4, and β6-α5). The guanine nucleobase of GDP/GMPPNP forms hydrogen bonds with the side chain carboxylate of Asp124, side chain of Asn121, and the main chain NH’s of Val152 and Lys153 (Fig. 2, *D*–*F*). The ribose of the guanosine forms a hydrogen bond with the side chain of Lys122 through its 4′O atom (Fig. 2, *D*–*F*). The α- and β-phosphates of GDP/GMPPNP form extensive hydrogen bonding networks with Ala19, Gly21, Lys22, Ser23, and Ser24 from the P-loop (Fig. 2, *G*–*I*).

### Inactivated state of hRab23

**Figure 3:**
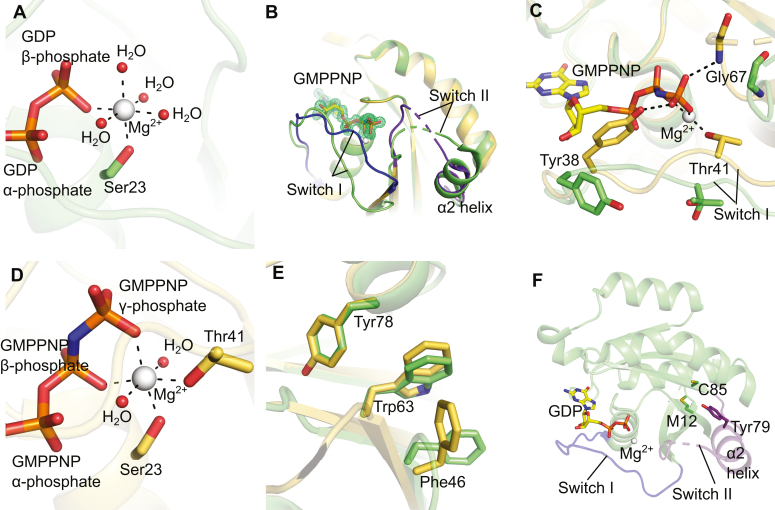
**Conformational changes of WT hRab23**_**7-172**_**.***A*, view of the octahedral coordination of the Mg^2+^ ion in the hRab23_7-172_–GDP structure (PDB ID 8YL3). *B*, conformational differences of switches I and II of hRab23_7-172_ shown through the superimpositioning of a hRab23_7-172_–GDP structure (*light green*) (PDB ID 8YL3) with a hRab23_7-172_-GMPPNP structure (*yellow orange*) (PDB ID 8YIM). Switch I (*blue*) and switch II (*purple*) of hRab23_7-172_-GMPPNP (PDB ID 8YIM) are highlighted; GMPPNP is shown in sticks; GDP not shown; *green* mesh represents the mF_o_-DF_c_ OMIT map of the GMPPNP ligand (contour = 3*σ*). *C*, conformational changes of residues Tyr38, Thr41, and Gly67 between the hRab23_7-172_-GDP structure (PDB ID 8YL3) (*light green*) and the hRab23_7-172_-GMPPNP structure (PDB ID 8YIM) (*yellow orange*). GMPPNP shown in *yellow* sticks, GDP not shown. *D*, view of the octahedral coordination of the Mg^2+^ ion in a hRab23_7-172_-GMPPNP structure (PDB ID 8YIM). *E*, superimposition of the hydrophobic triad (Phe46, Trp63, and Tyr79) from the structure of hRab23_7-172_-GDP (*light green*) (PDB ID 8YL3) onto the hRab23_7-172_-GMPPNP structure in *P*3_1_ 2 1 space group (*yellow orange*) (PDB ID 8YIM). *F*, positions of the residues Met12, Cys85, and Tyr79 that are mutated in Carpenter Syndrome (sticks) of the hRab23_7-172_–GDP complex (PDB ID 8YL3). Switch I (*blue*); switch II (*purple*); GDP/GMPPNP in sticks (C, *yellow*; O, *red*; N, *blue*; P, *orange*); Mg^2+^ in *white* sphere; water molecule in *red* spheres; hydrogen bond or electrostatic interactions are indicated by *black* dashes.

The structure of hRab23_7-172_ in complex with GDP (PDB ID 8YL3) confers the OFF state, as expected for this superfamily of small GTPases. When GDP is bound, the switch I loop adopts an opened conformation, resulting in the diphosphate of GDP not fully enclosed by the protein (Figs. 2*A* and S4*A*). Lys40 from switch I forms a hydrogen bond through its side chain with the 2′O of the GDP ribose. The main chain NH of Lys40 is hydrogen bonded to an O of the GDP α-phosphate (Fig. 2*D*). The hRab23_7-172_-GDP switch II region contains a loop with a 3-turned α-helix (α2-helix); however, two residues (Gln68 and Glu69) in switch II are disordered (Fig. 2*A*). An Mg^2+^ ion is octahedrally coordinated by an O of the β-phosphate of GDP, the O of the side chain hydroxyl of Ser23, and O atoms of four water molecules (Fig. 3*A*).

The hRab23_7-172_-GDP structure (PDB ID 8YL3) has high structural similarities with the mRab23-GDP structures (RMSDs of 129 C_α_ 0.38 (PDB ID 1Z2A) and 138 C_α_ 0.34 (PDB ID 1Z22)) (23). Overall, the hRab23_7-172_ -GDP structure superimposes well with the mRab23 structures with only slight variations in positions of the switch I or II regions (Fig. S1, *A* and *B*). The structural differences in the switch regions between the human and mouse proteins could be due to the flexible nature of these loops in the absence of a binding partner or differences in crystal packing.

### Activated state of hRab23

The two structures of the hRab23_7-172_–GMPPNP complexes (PDB IDs 8YIM and 8YNR) are very similar (RMSD of 128 C_α_ = 0.22) (Fig. S2). Both the GMPPNP-bound hRab23_7-172_ structures have similar switch I and II conformations (Fig. S2) but when compared with the hRab23_7-172_-GDP structure, the switch I and II regions of the GMPPNP-bound hRab23_7-172_ are conformationally different (Fig. 3*B*). In the presence of GMPPNP, the main chain NH of Gly67 in the switch II loop interacts with an O of the γ-phosphate of GMPPNP through a hydrogen bond (Fig. 3, *B* and *C*). This interaction leads to the switch II loop being pulled closer to the GMPPNP ligand, potentially leading to a change in the conformation of switch II, including the loss of a turn in the α2-helix within switch II. Nevertheless, the loops of switch II in both the GMPPNP-bound hRab23_7-172_ structures are still partially disordered (residues 69–73 for the one in space group *P*3_1_ 2 1 and residues 68–73 for the one in space group *P*2 2_1_ 2_1_). The repositioning of the switch II loop also appeared to induce conformational changes through the Interswitch region (between switch I and switch II). The switch I loop adopts a closed conformation in the structure of hRab23_7-172_-GMPPNP (ON state). In both the GMPPNP-bound Rab23 structures, the triphosphate of GMPPNP is fully enclosed by residues from the switch I and P (β1-α1) loops (Figs. 2, *B* and *C* and S4, *B* and *C*). Residues Tyr38 and Thr41 from switch I differed significantly in terms of their positions in the ON state relative to the OFF state (Fig. 3*C*). In the ON state, the side chain hydroxyl of Tyr38 from switch I formed a hydrogen bond with the γ-phosphate of GMPPNP while the side chain of Thr41 chelated Mg^2+^ trans to the β-phosphate (Fig. 3, *C* and *D*). The other five octahedral coordination sites of Mg^2+^ were occupied by the γ-phosphate O, β-phosphate O, side chain OH of Ser23, and two water molecules (Fig. 3*D*). On the other hand, in the OFF state, the side chains of Tyr38 and Thr41 pointed away from the nucleotide and Mg^2+^ ion and were more solvent-exposed (Fig. 3*C*).

We also compared the rotameric conformations of the hydrophobic triad (Phe46, Trp63, and Tyr78) of hRab23_7-172_ between the ON and OFF states. The hydrophobic triad is conserved among the Rab proteins (Fig. 1) and they are important in complementarity of effector binding (26). In the ON state, Phe46 adopted a different rotamer relative to the OFF state (∼90° rotation of the Cα-Cβ bond), which resulted in a T-shaped π stacking between Phe46 and Trp63 (Fig. 3*E*).

### Structures of the Carpenter syndrome–associated Rab23 Y79del mutant

**Figure 4:**
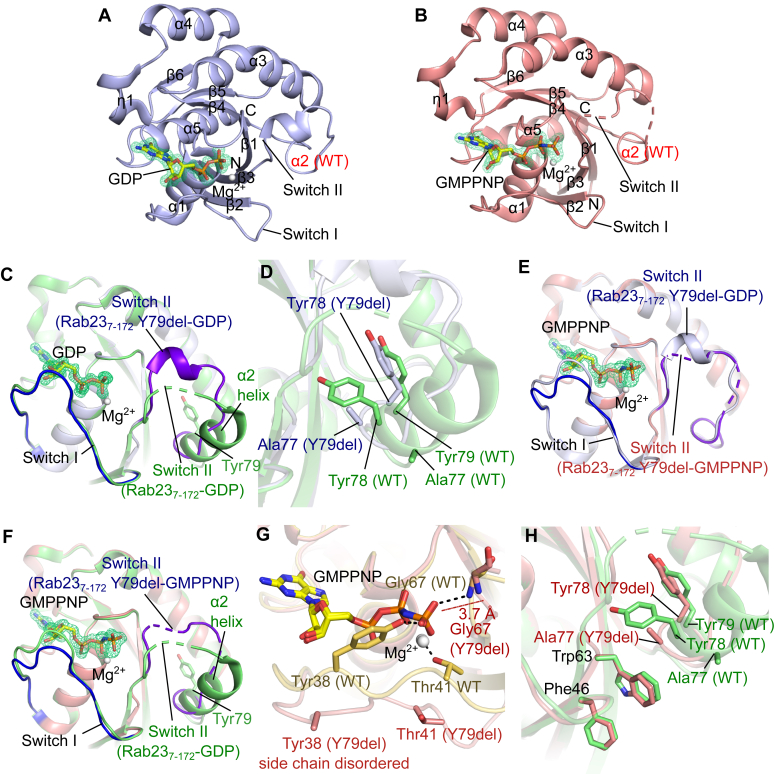
**Crystal structures of the hRab23**_**7-172**_**Y79del mutant in complex with GDP or GMPPNP.** Overall views of the structures of (*A*) hRab23_7-172_Y79del-GDP (PDB ID 8YO0) and (*B*) hRab23_7-172_Y79del-GMPPNP (PDB ID 8YP0). Supposed positions of the *α*2-helix from the WT hRab23_7-172_ are indicated with *red* fonts. Note that in the structures of the hRab23_7-172_ Y79del mutant, this region forms a random coil. *C*, structural comparisons of switches I and II between hRab23_7-172_-GDP (*light green*) (PDB ID 8YL3) and hRab23_7-172_Y79del-GDP (*light blue*) (PDB ID 8YO0). Switch I (*blue*) and switch II (*purple*) of hRab23_7-172_ Y79del-GDP (PDB ID 8YO0) are highlighted. GDP from hRab23_7-172_Y79del-GDP (PDB ID 8YO0) is shown in sticks. *D*, view of the region around the deleted Tyr79 in the Rab23 Y79del mutant. The structure of hRab23_7-172_ Y79del-GDP (*light blue*) (PDB ID 8YO0) is superimposed onto the structure of Rab23_7-172_-GDP (*light green*) (PDB ID 8YL3). Note that Tyr78 of hRab23_7-172_ Y79del-GDP (PDB ID 8YO0) occupies the position where Tyr79 from WT hRab23_7-172_-GDP (PDB ID 8YL3) is located. *E*, structural comparisons of switches I and II between hRab23_7-172_Y79del-GMPPNP (*salmon*) (PDB ID 8YP0) and hRab23_7-172_ Y79del-GDP (*light blue*) (PDB ID 8YO0). *F*, structural comparisons of switches I and II between hRab23_7-172_Y79del-GMPPNP (*salmon*) (PDB ID 8YP0) and hRab23_7-172_-GDP (*light green*) (PDB ID 8YL3). Switch I (*blue*) and switch II (*purple*) of hRab23_7-172_ Y79del-GMPPNP (PDB ID 8YP0) are highlighted. *G*, nucleotide-binding site of hRab23_7-172_-Y79del-GMPPNP (PDB ID 8YP0) (*salmon*) superimposed onto that of hRab23_7-172_-GMPPNP (PDB ID 8YIM) (*yellow orange*). Note that Gly67 of hRab23_7-172_Y79del-GMPPNP (*salmon*) (PDB ID 8YP0) is not close enough (maroon dashes; 3.7 Å) to form a hydrogen bond with the *γ*-phosphate of GMPPNP. *H*, superimposition of the hydrophobic triad (Phe46, Trp63, and Tyr79) region from the structures of hRab23_7-172_-GDP (PDB ID 8YL3) (*light green*) and hRab23_7-172_ Y79del-GMPPNP (PDB ID 8YP0) (*salmon*). GDP/GMPPNP are shown in *sticks* (C, *yellow*; O, *red*; N, *blue*; P, *orange*); Mg^2+^ in *white* spheres; *green* mesh represents the mF_o_-DF_c_ OMIT map of the GDP/GMPPNP ligand (contour = 3*σ*); hydrogen bond or electrostatic interactions are indicated by *black dashes*.

Several Rab23 mutants are clinically identified to be associated with Carpenter syndrome: three point mutations, M12K, C85R, and Y79 deletion (Y79del), are situated in the GTPase domain (Figs. 1 and 3*F*) (10). Other mutations include premature stop codons that would result in truncated proteins or failure of expression due to nonsense-mediated mRNA decay (10, 27). To elucidate the mechanisms of pathogenesis of the clinical mutants situated in the GTPase domain, we sought to produce recombinant hRab23_7-172_ clinical mutants, *that is*, M12K, C85R, and Y79del. However, the M12K and C85R mutants were insoluble when overproduced in *Escherichia coli* under our tested conditions (Fig. S3). Nevertheless, we successfully produced soluble hRab23_7-172_ Y79del mutant protein and crystalized it for X-ray diffraction studies. We determined a hRab23_7-172_ Y79del structure in complex with GDP (resolution 1.30 Å) (PDB ID 8YO0) (Fig. 4*A*). Like the WT hRab23_7-172_ structures, the GDP was copurified from *E. coli*. To explore the potential conformational changes of hRab23_7-172_ Y79del when complexed with GTP, we performed nucleotide exchange of hRab23_7-172_ Y79del with GMPPNP (a stable GTP analog) and crystalized the complex. We then determined a structure of hRab23_7-172_ Y79del in complex with GMPPNP (resolution 1.89 Å) (PDB ID 8YP0) (Fig. 4*B*).

The structure of the hRab23_7-172_ Y79del–GDP complex (PDB ID 8YO0) appeared to be largely similar to the WT hRab23_7-172_-GDP structure (PDB ID 8YL3) (RMSD of 131 C_α_ 0.274). The switch I loop of the hRab23_7-172_ Y79del-GDP structure adopted an opened conformation, similar to that in the WT hRab23_7-172_-GDP structure (Fig. 4*C*). Like the WT hRab23 protein, the Y79del mutant of hRab23_7-172_ copurified with GDP, as evidenced by clear observation of the electron density of GDP (Fig. 4, *A* and *C*). This observation suggests that the Y79 deletion does not abolish GDP binding. The switch II region of the GDP-bound hRab23_7-172_ Y79del, however, exhibited a different conformation (Fig. 4*C*) relative to that of the WT hRab23_7-172_-GDP structure. The switch II region of the hRab23_7-172_ Y79del mutant was more ordered than that of the WT Rab23_7-172_ structure. The partially disordered region (residues 67–70) observed in the WT hRab23_7-172_-GDP structure formed a 3_10_ helix in the hRab23_7-172_ Y79del mutant structure (Fig. 4*C*). Tyr79, whose side chain was buried in a hydrophobic pocket around the C-terminal ends of strands β1 and β3, originated from the α2 helix (part of Switch II) as observed in the WT hRab23_7-172_-GDP structure (Fig. 3*D*). However, in the hRab23_7-172_ Y79del-GDP structure, the residues corresponding to the α2 helix in WT hRab23_7-172_ formed a random coil instead of an α-helix (Fig. 4*C*), suggesting that the loss of Tyr79 potentially disrupts the secondary structure of the said region. On closer inspection of the mutation site, the loss of Tyr79 resulted in Tyr78 being repositioned to take the place of Tyr79 in the hydrophobic pocket while neighboring Ala77 moved to the original (WT) position of Tyr78 (Fig. 4*D*). Notably, the occupation of Ala77 in the original position of Tyr78, which is a residue in the hydrophobic triad, potentially altered the interaction surface with binding partners.

We then analyzed the hRab23_7-172_ Y79del-GMPPNP structure (PDB ID 8YP0) and discovered that the conformations of switches I and II were similar to those observed in the hRab23_7-172_ Y79del-GDP structure (PDB ID 8YO0) (Fig. 4*E*), despite the presence of GMPPNP in the nucleotide-binding pocket (Fig. 4, *B* and *E*). The switch I loop of hRab23_7-172_ Y79del-GMPPNP remained in the opened conformation, similar to that of the WT hRab23_7-172_ GDP-bound structure (PDB ID 8YL3) (Fig. 4*F*), suggesting that it remained in the OFF state. Residues in the switch I loop (*e.g.* Tyr38 and Thr41) that underwent major conformational changes upon activation remained in the OFF state positions instead (facing away from the nucleotide and Mg^2+^ ion) (Fig. 4*G*). Moreover, the switch II loop of hRab23_7-172_ Y79del-GMPPNP did not appear to fully engage the γ-phosphate of GMPPNP *via* the main chain NH of Gly67 (distance 3.7 Å; too far for an effective hydrogen bond) (Fig. 4*G*). Residues Trp63 and Phe46 of the hydrophobic triad in the structure of the hRab23_7-172_ Y79del–GMPPNP complex also appeared to adopt similar rotameric conformations as those in the WT hRab23_7-172_-GDP–bound structures (Fig. 4*H*). Overall, our structural data suggest that the hRab23_7-172_ Y79del mutant protein, despite having the ability to bind GMPPNP, failed to adopt an activated conformation thus likely to remain in the OFF state. This observation could imply potential impairment of hRab23 with the Y79del mutation in binding interactors, such as effectors and GAP, which requires hRab23 to be in the ON state, such as effectors and GAP.

### hRab23 Y79del has impaired GEF- and GAP-stimulated activities

To investigate the mutational effects of the Y79 deletion on hRab23 on its GTPase activities, we performed the GTPase-Glo assay (28), which measures the remaining GTP in the reaction. In this assay, the remaining GTP will be converted to ATP that gives rise to luminescence, which can be detected and quantified. We used the GTPase-Glo assay to measure the hRab23_7-172_ intrinsic GTPase activities and in the presence of the Intu–Fuz complex, which is the GEF of Rab23 (15, 20), and EVI5L, identified as a GAP of hRab23 (19).

**Figure 5:**
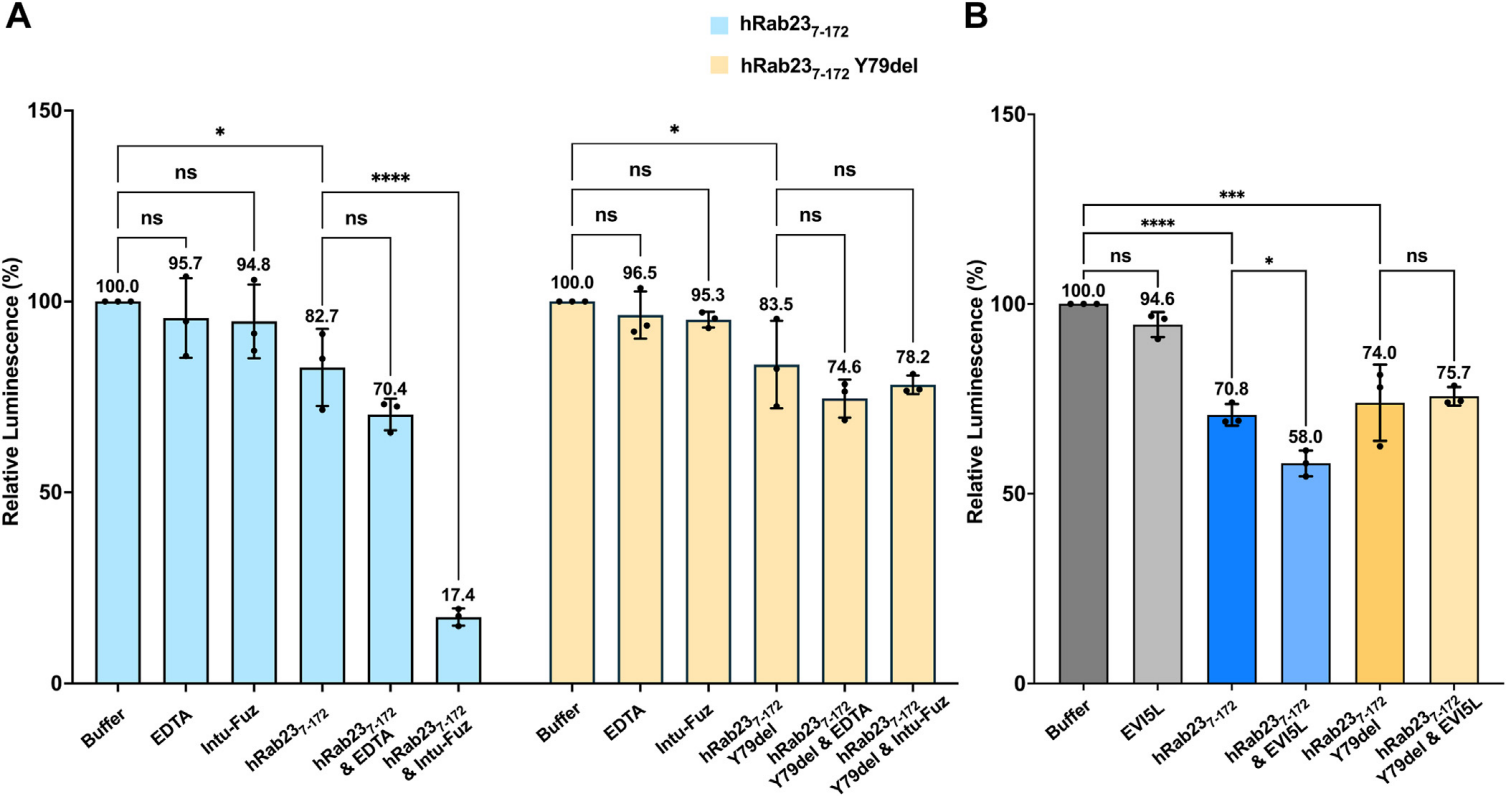
***In vitro* GTPase-Glo assay of hRab23**_**7-172**_**and hRab23**_**7-172**_**Y79del.***A*, GEF-aided nucleotide exchange activities. Heights of the bar graphs indicate the mean (n = 3; assay triplicates); *black dots* indicate individual data points; error bars indicate SD of the mean (n = 3; assay triplicates). ∗*p* < 0.05, ∗∗*p* < 0.01, ∗∗∗*p* < 0.001, ∗∗∗∗*p* < 0.0001, ns = not significant (two-way ANOVA followed by the Holm-Šídák test). *B*, GAP-stimulated GTPase activities. Heights of the bar graphs indicate the mean (n = 3; assay triplicates); *black dots* indicate individual data points; error bars indicate SD of the mean (n = 3; assay triplicates). ∗*p* < 0.05, ∗∗*p* < 0.01, ∗∗∗*p* < 0.001, ∗∗∗∗*p* < 0.0001, ns = not significant (one-way ANOVA followed by the Holm-Šídák test). Exact *p* values for the comparisons can be found in Table S3.

To investigate the effects of Y79 deletion on hRab23 nucleotide exchange activities aided by GEF, we incubated the purified hRab23_7-172_ Y79del mutant protein with and without the Intu–Fuz complex (GEF) with GTP for 2 h at 30 °C before measuring the luminescence corresponding to the remaining GTP in the reaction. As controls, we also set up parallel reactions with buffer only, 20 mM EDTA only, WT hRab23_7-172_, WT hRab23_7-172_ with 20 mM EDTA, hRab23_7-172_ Y79del mutant with 20 mM EDTA, WT hRab23_7-172_ with the Intu–Fuz complex (GEF), and the Intu–Fuz complex only. Both the WT hRab23_7-172_ and the hRab23_7-172_ Y79del mutant proteins used in this assay were in the GDP-loaded states. The GTPase-Glo assay results showed that WT hRab23_7-172_ recorded 82.7 ± 10.1% of luminescence relative to buffer control (Fig. 5*A*), suggesting that WT hRab23_7-172_ has some intrinsic nucleotide exchange activity. The samples containing 20 mM EDTA and either WT hRab23_7-172_ or hRab23_7-172_ Y79del recorded a further average decrease in luminescence (70.4 ± 4.2% and 74.6 ± 5.0% respectively relative to buffer), which was expected as EDTA could aid nucleotide exchange by chelating Mg^2+^. However, in this experiment, the luminescence of samples containing both EDTA and hRab23_7-172_/hRab23_7-172_ Y79del did not show significant differences when compared with just the hRab23 proteins alone. The samples containing both WT hRab23_7-172_ and the Intu–Fuz complex showed marked decrease in luminescence (17.4 ± 2.3% relative to buffer control) (Fig. 5*A*), indicating that the nucleotide exchange activity of WT hRab23_7-172_ can be significantly stimulated by its GEF. On the other hand, the hRab23_7-172_ Y79del mutant alone showed similar decrease in luminescence (83.5 ± 11.5% relative to buffer control) as WT hRab23_7-172_ (Fig. 5*A*), suggesting that the mutant has a similar intrinsic nucleotide exchange activity as the WT protein. However, the hRab23_7-172_ Y79del mutant in the presence of Intu–Fuz did not show a significant decrease in luminescence signals (78.2 ± 2.4% relative to buffer control) compared to hRab23_7-172_ Y79del alone (83.5 ± 11.5% relative to buffer control) (Fig. 5*A*). This observation suggests that the GEF-stimulated nucleotide exchange activity of the hRab23_7-172_ Y79del mutant is impaired. The impairment could potentially be due to changes in secondary structure of the switch II region, such as the loss of the *α*2 helix in this region, because the *α*2 helices of Rab proteins have been shown by other structures of Rab–GEF complexes (*e.g.* Rab21/Rabex5, Rab35/DENND1B, and Rab8a/Rabin8) to be involved in GEF interactions (29, 30, 31) (Fig. S5).

To investigate the effects of the Y79 deletion on the GAP-induced GTPase hydrolysis activity of hRab23, we used the GTPase-Glo assay to measure the remaining GTP levels of reactions involving WT hRab23_7-172_ and hRab23_7-172_ Y79del with and without EVI5L (GAP). Similar to the GEF assay, the hRab23_7-172_ and hRab23_7-172_ Y79del proteins used in the GAP assays were GDP-loaded without being subjected to nucleotide exchange prior to the reactions. In this assay, the reaction buffer contained 20 mM EDTA for the chelation of Mg^2+^ to mimic GEF activity, thus aiding GTP/GDP nucleotide exchange. The results showed that WT hRab23_7-172_ (70.8 ± 2.8% relative to buffer control) and the Y79del mutant (74.0 ± 10.1% relative to buffer control) only showed similar levels of luminescence (Fig. 5*B*), suggesting that they have similar intrinsic GTP hydrolysis activities. However, in the presence of EVI5L, WT hRab23_7-172_ recorded luminescence signal levels (58.0 ± 3.4% relative to buffer control) that are lower than that of the WT hRab23_7-172_ only (70.8 ± 2.8% relative to buffer control) (Fig. 5*B*), suggesting that the GTPase activity of WT hRab23_7-172_ can be stimulated by its GAP. However, hRab23_7-172_ Y79del in the presence of EVI5L did not show significant difference in luminescence (75.7 ± 2.4% relative to buffer control) when compared with hRab23_7-172_ Y79del only (74.0 ± 10.1% relative to buffer control) (Fig. 5*B*), suggesting that the hRab23_7-172_ Y79del mutant lost its GAP-stimulated GTPase activity. This loss in GAP-stimulated GTPase activity could be due to the failure of the mutant in adopting an ON state conformation despite the presence of GTP in the nucleotide-binding site, as evidenced by the structure of the hRab23_7-172_ Y79del–GMPPNP complex (PDB ID 8YP0) that we determined. This is further supported by structures of other Rab–GAP complexes (*e.g.* Rab33b/Gyp1-TBC-domain and Rab1b/TBC20) showing that Rab proteins interact with GAPs through both the switch I and II regions in the ON state (32, 33) (Fig. S6).

### Rab23 Y79del mutant loses functions in cells

**Figure 6:**
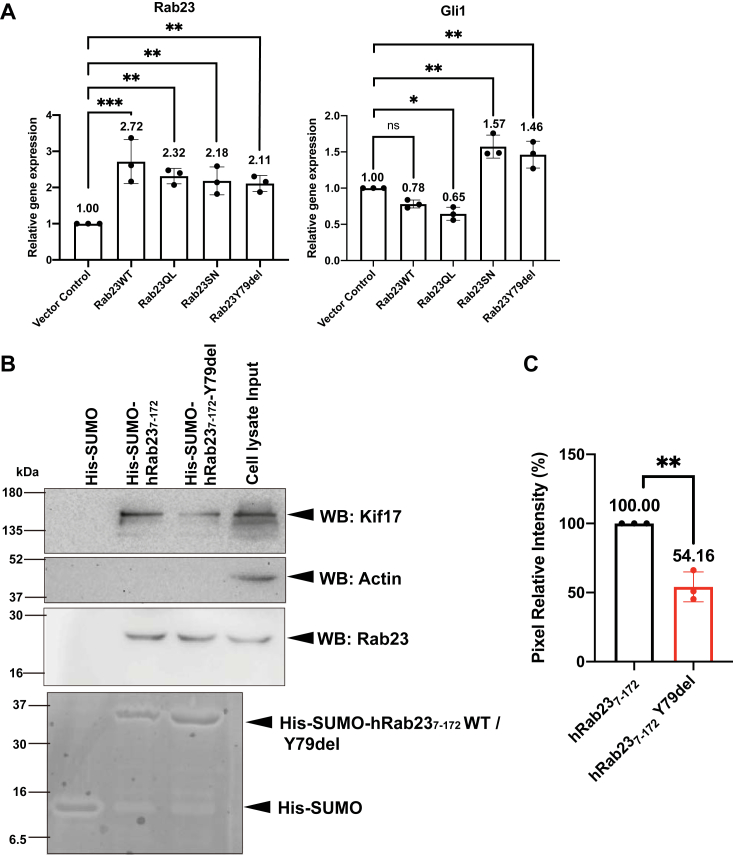
**Rab23**_**7-172**_**Y79del mutant lost the ability to repress Shh signaling pathway and less stably interacts with its functional interacting protein partner, KIF17.***A*, bar graphs depict the mean relative gene expression levels of *Rab23* and *Gli1* in NIH3T3 cells transduced with lentiviral carrying mouse Rab23 overexpression constructs (Rab23WT, Rab23QL, Rab23SN, Rab23Y79del). Data points represent mean ± S.D. from three independent experiments, each performed in triplicate. ∗∗ *p*-value ≤ 0.01, ∗ *p*-value ≤ 0.05, n.s. = not significant, two-way ANOVA followed by Tukey’s multiple comparisons test. Error bars depict ± S.D. *B*, representative Western blot showing a pull-down assay detecting the interaction of KIF17 and Rab23 proteins. His-SUMO-, His-SUMO-hRab23_7-172_-, and His-SUMO-hRab23_7-172_ Y79del-bound magnetic beads were incubated with lysates from HEK293 cells transfected with KIF17–mCitrine and treated with GMPPNP. The presence of KIF17, Rab23, and actin was assessed by Western blotting using specific antibodies against KIF17, Rab23, and Actin. A total of 20 μg of lysate was loaded as input and the levels of His-SUMO-, His-SUMO-hRab23_7-172_-, and His-SUMO-hRab23_7-172_ Y79del proteins utilized in the affinity pull-down were visualized on a Coomassie blue-stained gel, shown in the *bottom* panel. *C*, bar graph depicts the mean densitometric analysis of the KIF17 protein bands in the indicated pulldown groups. (n = 3 independent experiments) ∗∗ *p*-value ≤ 0.01, Unpaired 2-tailed Student’s *t* test. Error bars depict ± S.D. Exact *p* values for the comparisons can be found in Table S4.

To investigate if the Rab23 Y79del mutant loses its function in cells, we examined its effects on the Shh signaling pathway, which is negatively regulated by Rab23. Given that the Gli1 transcription factor is the downstream transcriptional target and effector of the Shh signaling pathway, the expression levels of *Gli1* reflect the levels of the Shh signaling pathway activation, which reflect the activities of Rab23 (3, 34). To achieve this, lentiviruses carrying transgenes were used to transduce NIH3T3 cells for the overexpression of different variants of mouse Rab23: Rab23 WT, the Rab23 Y79del mutant, Rab23QL (Q68L point mutation, a constitutive active (GTP-bound) mutant), as well as Rab23SN (S23N point mutation), the dominant negative mutant (GTP-binding impaired), respectively. The control group was transduced by lentiviruses carrying an empty vector. On day six after the viral transduction, the expression levels of *Rab23* and *Gli1* transcripts were examined by quantitative real-time PCR (qPCR). qPCR results showed that the expression of *Rab23* was significantly upregulated across all overexpression groups compared to the empty vector control group (Fig. 6*A*), indicating that the transgenes were successfully delivered and overexpressed in the NIH3T3 cells. In the Rab23QL overexpression group, the qPCR results showed that the *Gli1* expression was downregulated compared to the empty vector control (Fig. 6*A*), suggesting that Rab23 negatively mediates Shh signaling activity in the NIH3T3 cells. On the other hand, the overexpression of the Rab23 Y79del and Rab23SN mutants significantly upregulated the expression levels of *Gli1* as compared to that of the vector control group (Fig. 6*A*), reflecting an ectopic activation of the Shh signaling activity. Given that the overexpression of the Y79del mutant led to an increased activation of the Shh signaling activity (rather than unchanged activity compared to the control group), this observation suggests that the presence of the Y79del mutant protein has disrupted the capacity of the WT Rab23 in suppressing the Shh signaling pathway. Therefore, the results imply that the Y79del mutant, similar to that of the Rab23SN dominant negative mutant, exerts a dominant negative function in regulating the Shh signaling pathway.

Previous work demonstrated that Rab23 physically interacts with KIF17 to mediate the ciliary transport of KIF17 (21, 35). Informed by our determined crystal structure of the hRab23_7-172_ Y79del–GMPPNP complex (PDB ID 8YP0), we speculate that the Rab23 Y79del mutant would not be able to stably interact with KIF17 given its inability to adopt the active GTP-bound conformation. To test the interactions between Rab23 and KIF17, a pull down assay was performed in the lysates of HEK293T cells overexpressed with KIF17–mCitrine using His-SUMO-tagged WT hRab23_7-172_ and His-SUMO-tagged hRab23_7-172_ Y79del recombinant proteins purified from *E. coli* and immobilized on Dynabeads His-tag Isolation and Pulldown magnetic beads. The His-SUMO-tagged WT hRab23_7-172_ and His-SUMO-tagged hRab23_7-172_ Y79del proteins were previously exchanged with GMPPNP by *in vitro* nucleotide exchange, to enable the ON state of Rab23 for complexation with KIF17. Immunoblotting analyses of the pull down showed that the signal of KIF17 in the hRab23_7-172_ Y79del mutant pull down sample was markedly reduced when compared with that of the WT hRab23_7-172_ pull down sample (Fig. 6, *B* and *C*). The results are in line with our speculation, that the hRab23_7-172_ Y79del mutant exerted diminished interactions with KIF17 relative to the WT hRab23_7-172_, despite the presence of GMPPNP. Taken together, our findings from both the *in vitro* functional assays suggest that the hRab23_7-172_ Y79del mutant lost its GTP/GDP cycling-dependent activities and functions.

## Discussion

Rab23 is an important small GTPase for membrane trafficking. Disruption of its function is a key contributing factor in developmental anomalies presented by Carpenter syndrome. Our structural data provided insights into the conformation of the activated and inactivated forms of hRab23_7-172_. These two conformations are similar to those of other small GTPases whereby the switch I loop adopts the closed conformation when GMPPNP (GTP analog) is bound and opened conformation when GDP is bound. In addition to the WT hRab23_7-172_ structures, we have also determined structures of the hRab23_7-172_ Y79del mutant, a clinical mutant found in some individuals with Carpenter syndrome (10), in complex with GDP and GMPPNP, respectively. The structure of the hRab23_7-172_ Y79del mutant in complex with GDP showed that the switch II loop conformation differs from that of WT hRab23_7-172_, particularly in the region near the *α*2 helix and Tyr78, which forms part of the hydrophobic triad. The corresponding region has been shown by reported structures of other Rab–GEF complexes to be involved in GEF binding (29, 30, 31). Therefore, local structural changes because of the Y79 deletion could affect Intu–Fuz (GEF) binding to hRab23. Biochemical investigations using an *in vitro* GTPase assay demonstrated that the hRab23_7-172_ Y79del mutant lost its GEF-stimulated nucleotide exchange activity, appearing to be consistent with our structural findings.

A previous report postulated that the Rab23 Y79del mutant most likely loses its ability to bind effector(s) (10). Our structural data showed that the Y79del mutant when complexed with GMPPNP still adopted the OFF conformation, therefore supporting the postulation that the Y79del clinical mutant loses its ability to bind effector(s). Our attempts to produce recombinant KIF17 using the *E. coli* expression system were unsuccessful. Therefore, we were not able to test the interactions between Rab23 and KIF17 *in vitro* using recombinant proteins. Nevertheless, we showed that the His-SUMO-Rab23 Y79del-GMPPNP complex pulls down less KIF17 protein from HEK293T cell lysates when compared to the WT hRab23–GMPPNP complex, partially supporting the above postulation that the Y79del mutant loss its ability to bind effectors. Cellular functional assays showed that overexpression of the Rab23 Y79del mutant failed to negatively regulate the Shh signaling pathway, resulting in *Gli1* levels that were similar to cells with overexpressed dominant negative mutant, Rab23SN, providing evidence that the Y79del mutant exhibits a dominant negative function in cells. Additionally, our *in vitro* GTPase assay also showed that hRab23_7-172_ Y79del lost its GAP-stimulated activity. The inability of the mutant in adopting an activated complex conformation could also disrupt GAP binding, as supported by structures of other Rabs in complexes with respective GAPs (32, 33).

Under our *in vitro* GTPase assay conditions, we observed that the WT hRab23_7-172_ showed higher GEF-aided activities than GAP-stimulated activities. While the GEF and GAP assays were carried out in slightly different conditions and could not be directly compared, results from the GEF assay samples containing 20 mM EDTA and WT hRab23_7-172_ or the Y79del mutant, which recorded only slight decrease in luminescence relative to the proteins alone, suggests that EDTA is not as effective as Intu-Fuz in aiding nucleotide exchange. Therefore, the relatively lower GAP-stimulated activities of WT hRab23_7-172_ relative to its GEF-aided activities could be attributed to the low efficiency in nucleotide exchange by 20 mM EDTA in the GAP buffer. There were also slight average decreases, though not statistically significant, in luminescence (<6%) relative to buffer in the Intu-Fuz and EVI5L-only controls. We speculate that these result from minor changes in the reaction conditions attributed to the presence of a small amount of storage buffers containing the Intu-Fuz and EVI5L proteins. Additional test GEF and GAP GTPase assays carried out by adding the corresponding volumes of buffers from the Intu-Fuz and EVI5L proteins (without proteins) showed marginal average decreases in luminescence relative to the reaction buffers alone (97.4% and 98.4%, though similarly not statistically significant) (Fig. S7), suggesting that the fluctuations in luminescence observed in the Intu-Fuz and EVI5L-only samples were likely not caused by GEF or GAP protein activities.

Combining all our data, we conclude that the hRab23_7-172_ Y79del mutant lost its GEF- and GAP-stimulated activities and likely also unable to bind effectors even if GTP is able to occupy the active site (a possibility through intrinsic nucleotide exchange activities). Therefore, the hRab23 Y79del mutant is nonfunctional thus leading to the Carpenter syndrome phenotype.

## Experimental procedures

### Molecular cloning and site-direct mutagenesis

An N-terminally hexahistidine-SUMO tagged human Rab23 residues 7 to 172 (pET28a-His-SUMO-hRab23_7-172_) was cloned into pET28a vector by transfer PCR (36). The codon-optimized genes of human Fuzzy, Inturned, and EVI5L were chemically synthesized (GenScript). The genes encoding for Inturned and EVI5L were cloned into a pFastBac-HT-B vector (containing an N-terminal hexahistidine tag, followed by a TEV protease cleavage site) and the gene encoding for Fuzzy was cloned into a pFasBac-Dual vector for baculovirus expression (GenScript).

The hRab23_7-172_ M12K, C85R, and Y79del mutants for structural studies were generated by site-directed mutagenesis using pET28a-His-SUMO-hRab23_7-172_ as templates, in which QuickChange mutagenesis was used for M12K and C85R (37, 38), whereas transfer PCR (36) was used for Y79del. Full-length mouse Rab23 (mRab23) with Y79del mutation for functional studies were also generated by QuickChange mutagenesis (37, 38). The DNA primers used can be found in Table S1.

### Small-scale expression trials of hRab23_7-172_ mutants

*E. coli* BL21 (DE3) cells transformed with respective pET28a-His-SUMO-hRab23_7-172_, pET28a-His-SUMO-hRab23_7-172_ M12K, pET28a-His-SUMO-hRab23_7-172_ C85R, pET28a-His-SUMO-hRab23_7-172_ Y79del plasmids were cultured at 37 °C in 5 ml LB media supplemented with a final concentration of 50 μg/ml kanamycin. When the A_600_ reached 0.4, protein expression was induced by adding a final concentration of 50 μM IPTG. The cells were allowed to grow for a further 16 h at 22 °C before being harvested by centrifugation, resuspended in lysis buffer (20 mM Tris, pH 7.5, 150 mM NaCl, 5 mM MgCl_2_, 5 mM imidazole, 5 mM β-mercaptoethanol (BME), and 1 mM PMSF), sonicated, and centrifuged. The supernatant was incubated with 20 μl slurry of Ni-NTA resins (Macherey-Nagel) for 1 h. The Ni-NTA beads were then washed twice with 100 μl of wash buffer (20 mM Tris, pH 7.5, 500 mM NaCl, 5 mM MgCl_2_, 25 mM imidazole, and 5 mM BME) and the proteins were eluted by adding 20 μl of elution buffer (20 mM Tris, pH 7.5, 500 mM NaCl, 5 mM MgCl_2_, 250 mM imidazole, and 5 mM BME) to the beads. The cell debris (pellet), soluble lysates, and eluates were analyzed by 15% SDS-PAGE.

### Production and purification of hRab23_7-172_ and hRab23_7-172_ Y79del

The plasmids pET28a-His-SUMO-hRab23_7-172_ and pET28a-His-SUMO-hRab23_7-172_ Y79del were transformed into *E. coli* BL21 (DE3) competent cells and grown at 37 °C in 2xYT media supplemented with a final concentration of 50 μg/ml kanamycin. The cells were induced with a final concentration of 50 μM IPTG when the A_600_ reached 0.4, cell growth was continued at 22 °C for 16 h. The cells were harvested by centrifugation, resuspended in lysis buffer (20 mM Tris, pH 7.5, 150 mM NaCl, 5 mM MgCl_2_, 5 mM imidazole, 5 mM BME, and 17.8 μg/ml PMSF), and sonicated. The lysate was supplemented with a final concentration of 0.5% Triton X-100 before being clarified by high-speed centrifugation. The supernatant was loaded onto a gravity column containing Ni-NTA agarose beads (Macherey-Nagel), The beads were washed with wash buffer (20 mM Tris, pH 7.5, 500 mM NaCl, 5 mM MgCl_2_, 5 mM BME, and 25 mM imidazole) and the protein was eluted with elution buffer (20 mM Tris, pH 7.5, 500 mM NaCl, 5 mM MgCl_2_, 5 mM BME, and 250 mM imidazole). The His-SUMO tag was cleaved by adding ULP1 protease and incubated at 4 °C overnight. Any residual uncleaved His-SUMO-tagged protein and the cleaved His-SUMO tag were removed by nickel affinity. The untagged proteins were further purified by size-exclusion chromatography using a HiLoad 16/600 Superdex 200 pg column (Cytiva) pre-equilibrated with a buffer containing 20 mM Tris, pH 7.5,150 mM NaCl, 5 mM MgCl_2_, and 5 mM DTT. The purified proteins were concentrated to ∼35 mg/ml, snap frozen in liquid nitrogen, and stored at −80 °C. For pull-down assays, the His-SUMO tag was not cleaved prior to purification by size-exclusion chromatography.

### Production and purification of the Intu-Fuz complex (GEF)

The production and purification of the Intu–Fuz complex were adapted from a previous report (39). Intu and Fuz were expressed in *Trichoplusia ni* (Hi-5) insect cells by coinfection with baculoviruses carrying the respective Intu and Fuz genes. The cells were harvested by centrifugation, resuspended in lysis buffer (20 mM Tris, pH 8.0, 250 mM NaCl, 5% glycerol, 5 mM MgCl_2_, and 10 mM imidazole) supplemented with protease inhibitor cocktail (Roche), 0.05% Triton X-100, 1 mM BME, and 17.8 μg/ml PMSF, sonicated, and centrifuged at high-speed. The supernatant was loaded onto a gravity column containing Ni-NTA resins (Macherey-Nagel), which were then washed with wash buffer (20 mM Tris, pH 8.0, 250 mM NaCl, 5% glycerol, 5 mM MgCl_2_, 1 mM BME, and 40 mM imidazole) and the proteins were eluted with elution buffer (20 mM Tris, pH 8.0, 250 mM NaCl, 5% glycerol, 5 mM MgCl_2_, 1 mM BME, and 350 mM imidazole). The His-tag was cleaved by the addition of TEV protease. The target proteins were then further purified using a HiTrap Q column (Cytiva) followed by a HiLoad 16/600 Superdex 200 pg column (Cytiva) pre-equilibrated with 20 mM Tris, pH 8.0, 200 mM NaCl, and 1 mM DTT. The purified Intu–Fuz complex was concentrated to 18 mg/ml, snap frozen in liquid nitrogen, and stored at −80 ˚C.

### Production and purification of EVI5L (GAP)

The protocol for the production and purification of full-length EVI5L is similar to those used for the Intu–Fuz complex, except that the buffer for the size-exclusion chromatography was 20 mM Tris, pH 7.5, 250 mM NaCl, and 1 mM DTT. The purified protein was concentrated to 14 mg/ml, snap frozen in liquid nitrogen, and stored at −80 °C.

### hRab23_7-172_ nucleotide-exchange

Nucleotide-exchange of Rab23 proteins was performed to replace GDP (co-purified with Rab23 proteins) with GMPPNP as reported (40) with some modifications. Three hundred ninety-four micromolars of hRab23_7-172_ protein, 3 U per mg of hRab23_7-172_ of calf intestinal alkaline phosphatase (CIP) (NEB), and 1.97 mM GMPPNP were added into a final volume of 500 μl buffer containing 25 mM Tris, pH 8.0, 0.1 mM ZnCl_2_, and 200 mM ammonium sulphate. The mixture was first incubated at 37 °C for 30 min, then supplemented with a final concentration of 20 mM MgCl_2_, before being subjected to size-exclusion chromatography using either a Superdex 200 Increase 10/300 Gl or a Superdex 75 Increase 10/300 Gl column (Cytiva) pre-equilibrated with 20 mM Tris, pH 7.5, 150 mM NaCl, 5 mM MgCl_2_, and 5 mM DTT. The nucleotide-exchanged hRab23_7-172_ proteins were pooled and concentrated to 35 mg/ml.

### Crystallization of hRab23_7-172_ and hRab23_7-172_ Y79del

Crystals of the hRab23_7-172_-GDP, hRab23_7-172_-GMPPNP in *P*2 2_1_ 2_1_ space group, and hRab23_7-172_ Y79del-GDP complexes were grown by hanging drop vapor diffusion. Equal volumes of 1 μl 15 mg/ml protein solution and reservoir solution were mixed and equilibrated over a 500 μL reservoir solution at 293 K. The hRab23_7-172_–GDP complex solution has an additional 20% dimethylsufloxide prior to mixing with the reservoir solution. The reservoir solution conditions can be found in Table S2.

Crystals of the hRab23_7-172_-GMPPNP in *P*3_1_ 2 1 space group and hRab23_7-172_ Y79del–GMPPNP complexes were grown by sitting drop vapor diffusion prepared by a Crystal Gryphon Dispenser (Art Robbins). Equal volumes of 150 nl 20 mg/ml protein solution and reservoir solution were mixed and equilibrated over a 60 μl reservoir solution at 293 K. The reservoir solution conditions can be found in Table S2.

The crystals were first soaked briefly in a cryoprotectant solution containing the reservoir solution diluted with a final concentration of 25% (v/v) glycerol before being harvested and flash cooled in liquid nitrogen.

### X-ray data collection and structure determination

X-ray data were collected at 100 K using in-house diffractometers (Rigaku FR-X) containing rotating anode sources (X-ray wavelength 1.5418 Å) and equipped with either a HyPix 6000C detector (for crystals of hRab23_7-172_-GDP, hRab23_7-172_-GMPPNP (two crystal forms), and hRab23_7-172_Y79del-GDP) or an EIGER-4M detector (crystal of hRab23_7-172_Y79del-GMPPNP). The data of hRab23_7-172_-GDP, hRab23_7-172_-GMPPNP (two crystal forms), and hRab23_7-172_Y79del-GDP were indexed, integrated, and scaled by CrysAlisPro (Agilent Technologies UK ltd) and merged by AIMLESS in CCP4 (41). The data for hRab23_7-172_Y79del-GMPPNP were indexed, integrated, scaled, and merged by XDS (42). The structures were solved by molecular replacement using Phaser (43). The original model used for molecular replacement search was the mouse Rab23 structure (PDB: 1Z2A) (23), which successfully provided a solution for the hRab23_7-172_ Y79del–GDP complex. The solutions for the other human Rab23 structures were searched by using subsequent human Rab23 structures as search models. The models were fitted and refined iteratively using COOT (44) and PHENIX.REFINE (45). The twin law -*h*, *l*, *k* was applied to the refinement of the structure of the hRab23_7-172_Y79del–GMPPNP complex (PDB ID 8YP0). Crystallographic data collection and refinement statistics can be found in Table 1.

**Table 1:** X-ray data collection and refinement statistics

Structural complex	hRab23_7-172_-GDP	hRab23_7-172_-GMPPNP 1	hRab23_7-172_-GMPPNP 2	hRab23_7-172_ Y79del- GDP	hRab23_7-172_ Y79del-GMPPNP
PDB ID	8YL3	8YIM	8YNR	8YO0	8YP0
Radiation source	Rigaku FRX	Rigaku FRX	Rigaku FRX	Rigaku FRX	Rigaku FRX
Detector	Hypix6000C	Hypix6000C	Hypix6000C	Hypix6000C	EIGER4M
X-ray wavelength, Å	1.5418	1.5418	1.5418	1.5418	1.5418
Resolution range^a^, Å	14.29–1.20 (1.24–1.20)	21.76–1.35 (1.40–1.35)	14.94–1.80 (1.86–1.80)	15.11–1.30 (1.35–1.30)	27.77–1.89 (1.95–1.89)
Space group	*P*2_1_ 2_1_ 2_1_	*P*3_1_ 2 1	*P*2 2_1_ 2_1_	*P*2_1_ 2_1_ 2_1_	*P*2_1_ 2_1_ 2_1_
Unit cell dimensions	*a* = 42.9 Å, *b* = 57.2 Å, *c* = 63.0 Å, α = β = γ = 90°	*a* = *b* = 60.5 Å, *c* = 93.9 Å, α = β = 90°, γ = 120°	*a* = 38.7 Å, *b* = 61.3 Å, *c* = 70.6 Å, α = β = γ = 90°	*a* = 40.8 Å, *b* = 51.6 Å, *c* = 74.6 Å, α = β = γ = 90°	*a* = 40.8 Å, *b* = 61.7 Å, *c* = 62.2 Å, α = β = γ = 90°
Total no. of reflections observed^a^	233,349 (18,417)	399,702 (30,103)	181,957 (17,379)	225,685 (18,990)	83,805 (7843)
No. of unique reflections^a^	49,045 (4850)	44,368 (4379)	16,127 (1587)	39,436 (3902)	13,114 (1242)
Multiplicity^a^	4.8 (3.9)	9.0 (6.9)	11.3 (11.0)	5.7 (4.9)	6.4 (6.3)
Completeness^a^, %	99.87 (99.98)	99.96 (100.00)	98.97 (95.72)	99.79 (100.00)	99.30 (95.54)
Mean I/σ(I)^a^	18.77 (1.61)	24.52 (2.09)	19.27 (1.56)	13.75 (1.69)	12.64 (1.81)
Wilson B-Factor, Å^2^	10.58	9.90	20.80	9.34	23.74
R_merge_^a^	0.04868 (0.8426)	0.1311 (1.378)	0.1082 (1.612)	0.08304 (0.9751)	0.1117 (1.402)
CC_1/2_^a^	1 (0.572)	0.997 (0.744)	0.999 (0.958)	0.999 (0.618)	0.998 (0.585)
R_work_^a^	0.1670 (0.2448)	0.1496 (0.1861)	0.2033 (0.3009)	0.1581 (0.2309)	0.1871 (0.2698)
R_free_^a^	0.1890 (0.2525)	0.1794 (0.2178)	0.2312 (0.3094)	0.1858 (0.2696)	0.2370 (0.2869)
No. of non-H atoms (Total)	1623	1518	1369	1498	1397
No. of non-H atoms (Macromolecules)	1363	1292	1231	1297	1254
No. of non-H atoms (Ligands)	29	33	33	29	33
No. of non-H atoms (Solvent)	231	193	105	172	110
Protein residues	164	160	160	165	160
RMS deviation (Bonds/Angles)	0.007 Å/1.32°	0.003 Å/0.54°	0.009 Å/0.99°	0.012 Å/1.31°	0.005 Å/0.70°
Ramachandran outliers, %	0.00	0.00	0.00	0.00	0.00
Average B-factor (All atoms), Å^2^	16.37	16.58	34.44	16.31	28.68
Average B-factor (macromolecules), Å^2^	14.14	14.91	34.67	14.64	28.42
Average B-factor (ligands), Å^2^	10.33	7.75	19.22	9.04	21.07
Average B-factor (solvent), Å^2^	30.25	29.26	36.49	30.15	33.92

R_free_ is based on 5.0% of the reflections used in refinement.

a Statistics for the highest-resolution shell are shown in parentheses.

### *In vitro* GTPase assay

The GTPase-Glo assay (Promega) (28) was used to measure the GTPase activities of hRab23 catalyzed by GEF (the Intu–Fuz complex) for nucleotide exchange and GAP (EVI5L) for accelerating the GTPase activities. The reactions were performed in triplicates using a 384-well plate (Corning costar), following the protocol provided by the manufacturer.

To assay for the GEF-aided nucleotide exchange activities, 10 μl reactions containing a final concentration of 3 μM hRab23_7-172_ WT or Y79del mutant protein (in the originally purified GDP-loaded form without nucleotide exchange), 2.25 μM Intu–Fuz complex, and 5 μM GTP were carried out in the GEF buffer (50 mM Tris-HCl, pH 7.5, 50 mM NaCl, 1 mM EDTA, and 10 mM MgCl_2_) provided by the manufacturer (Promega). To assay for the GAP-stimulated GTPase activities, 10 μl reactions containing 3 μM hRab23_7-172_ WT or Y79del mutant protein (in the originally purified GDP-loaded form without nucleotide exchange), 2.25 μM EVI5L, and 5 μM GTP were carried out in the GTPase/GAP buffer (50 mM Tris–HCl, pH 7.5, 50 mM NaCl, 20 mM EDTA, and 5 mM MgCl_2_) provided by the manufacturer (Promega). All the reactions were initiated by adding the GTP component to the reaction mixtures, followed by incubation at 30 °C for 2 h. To quench the reactions and generate ATP from the remaining GTP, 10 μl of the reconstituted GTPase-Glo reagent containing 1× GTPase-Glo reagent and 5 μM of ADP were added into each reaction mixture. The 384-well plate containing the reaction mixtures was further incubated at room temperature for 30 min. After that, 20 μl of Detection Reagent (Promega) was added into each reaction mixture, followed by another incubation at room temperature for 10 min. The luminescence signals were measured using a CLARIOstar^Plus^ microplate reader (BMG Labtech). Data were presented as mean ± SD. Statistical analyses for comparisons among groups were performed by one-way (GAP assay) and two-way (GEF assays) ANOVA followed by the Holm-Šídák test.

### Cell culture, expression vector, and viral transduction

Human HEK293 and mouse NIH3T3 cell lines were obtained from the American Type Culture Collection. Cell lines were tested for *mycoplasma* contamination by PCR. NIH3T3 and HEK293 cells were grown in Dulbecco's modified Eagle's medium (12800017, Gibco Life Technologies) supplemented with 10% fetal bovine serum (10270106, Gibco Life Technologies) and 1× penicillin-streptomycin (15140122, Gibco Life Technologies). All cells were maintained at 37 °C with 5% CO_2_ levels.

For the *in vitro* overexpression of Rab23 and different mutants in NIH3T3 cells, lentiviral gene delivery method was employed. FUGW lentivirus vectors carrying full-length mouse Rab23WT, Rab23SN, and Rab23QL were reported in our previous publication (46). The Rab23 Y79del mutant was obtained by performing site-direct mutagenesis on the FUGW-Rab23WT construct. The Ubc promoter was used to drive the expression in mammalian cells. KIF17–mCitrine was a kind gift from BL Tang.

For viral transduction, the lentiviruses were prepared according to previously described protocol (47). NIH3T3 were transduced on day 0 of cell seeding. Six days post-virus transduction, total RNA was extracted for reverse transcription into cDNA and quantitative PCR.

### Real-time qPCR

Total RNA was extracted from cells using an RNA extraction reagent (R40101, Vazyme). After determination of the RNA concentration, equal amount of RNA extracts were reverse transcribed into equal volumes of cDNA using the iScript cDNA Synthesis Kit (1708890, Biorad). The expression levels of the genes of interest were evaluated by performing qPCR using equal volumes of cDNA template across all samples. The qPCR assays were performed using TB Green Master Mix (RR820a, Takara) and CFX Opus 96 Real-Time PCR system (Biorad), following the manufacturer’s protocol. The Ct values for each target were normalized to the *Gapdh* gene to obtain the *Δ*Ct values. Relative gene expression level of the overexpression groups were calculated by the *ΔΔ*Ct method, normalizing to the vector control group. The PCR primers used were as follows: mouse *Gapdh*: F-5′-TTCACCACCATGGAGAAGGC-3′, R-5′-GGCATGGACTGTGGTCATGA-3′; mouse *Rab23*: F-5′-AGGCCTACTATCGAGGAGCC-3′, R-5′-TTAGCCTTTTGGCCAGTCCC-3′; mouse *Gli1*: F-5′-CCCATAGGGTCTCGGGGTCTCAAAC-3′, R-5′-GGAGGACCTGCGGCTGACTGTGTAA-3′. Statistical analyses for comparisons among groups were performed by two-way ANOVA followed by Tukey’s multiple comparisons test.

### His pull-down assay

For the pull-down experiment, His-SUMO-hRab23_7-172_ and His-SUMO-hRab23_7-172_ Y79del purified proteins were first subjected to nucleotide exchange with GMPPNP to form the GTP-analog–bound His-SUMO-hRab23_7-172_–GMPPNP and His-SUMO-hRab23_7-172_ Y79del–GMPPNP complexes, respectively. The complexes were purified by size-exclusion chromatography as detailed above (in the *hRab23*_*7-172*_ *nucleotide-exchange* Experimental Procedures section). Cell lysates of HEK293 cells overexpressed with KIF17 were prepared using standard lysis buffer containing 0.5% Triton X-100, with freshly added protease/phosphatase inhibitor cocktail (5872, Cell Signaling Technology) and 0.1 mM PMSF (P7626, Sigma-Aldrich). The cell lysate was added with 20 μM of GMPPNP and incubated at 4 °C for 1 h. To obtain the pull-down complexes, 1 μg of each purified protein samples, that is, His-SUMO, His-SUMO-hRab23_7-172_, and His-SUMO-hRab23_7-172_ Y79del, were respectively incubated with 10 μl of Dynabeads magnetic beads (10103D, Invitrogen) and 1 mg of HEK293 cell lysates overnight. After incubation, the beads were washed several times with lysis buffer. The his-tagged fusion proteins and its interacting partners were eluted by heating in SDS loading buffer. The proteins were resolved by 12.5% SDS-PAGE and transferred onto PVDF membranes. The membranes were incubated with the following primary antibodies: rabbit anti-Rab23 (Proteintech, 1:2000), mouse anti-Actin (DSHB, 1:5000), and rabbit anti-KIF17 (Proteintech, 1:2000). The membranes were further incubated with their respective HRP-conjugated secondary antibodies (1:5000). Antibody specificity was verified through visualization of the His-SUMO negative control sample. Immunoblotted proteins were visualized using ECL substrate (34577, Thermo Fisher Scientific) with a ChemiDoc MP Imaging System (Bio-Rad). The relative band intensities were quantified using the software ImageJ. Statistical analyses were performed by unpaired, 2-tailed Student’s *t* test.

## Data availability

Atomic coordinates and structure factors of the crystal structures presented in this article have been deposited with the Protein Data Bank under accession numbers 8YL3, 8YIM, 8YNR, 8YO0, and 8YP0.

## Supporting information

This article contains supporting information (23, 29, 30, 31, 32, 33).

## Conflict of interest

All authors declare that they have no conflicts of interests with the contents of this article.
