# Supplementary material for: Structural basis for Rab23 activation and a loss-of-function mutation in Carpenter syndrome
Source: J Biol Chem. 2024 Nov 29;301(1):108036. doi: 10.1016/j.jbc.2024.108036 (PMC11730874; doi:10.1016/j.jbc.2024.108036)
Supplement: Supporting information [file mmc1.pdf]

Structural basis for Rab23 activation and a loss-of-function mutation in Carpenter Syndrome

Yat Yin Chau[1], Hanbin Liang[1], Wai Lam Tung[1], Catherine Hong Huan Hor[1]*, Wei Shen Aik[1]*

[1] Department of Chemistry, Hong Kong Baptist University, Kowloon Tong, Hong Kong SAR, China.

*Corresponding authors. Email: aikweishen@hkbu.edu.hk (WSA); catherinehor@hkbu.edu.hk (CHHH)

Supporting Information:

Materials included:

Tables S1-S4

Figures S1-S7

Table S1. DNA primer sequences for cloning and site-directed mutagenesis

	Forward Primer (5′ to 3′)	Reverse Primer (5′ to 3′)
Cloning using Transfer PCR		
pET28a-His-SUMO-hRab23$_{7\text{-}172}$ wild type	GAGGCTCACAGAGAACAGATTGGTGGATCCGAGGTGGCGATTAAAATGGTTG	TCGGGCTTTGTTAGCAGCCGGATCTCAGTGTTACTTTTGCAGATACTTCTCCGC
QuickChange^{TM} Site-directed mutagenesis		
pET28a-His-SUMO-hRab23$_{7\text{-}172}$ M12K	CGATTAAAAAGGTTGTGGTTGGTAATGGCGCGGTTGGTAAAAGC	CAACCACAACCTTTTTAATCGCCACCTCGGATCCACCAATCTG
pET28a-His-SUMO-hRab23$_{7\text{-}172}$ C85R	CAAGCGCGCGTGCTGGTTTTCAGCACCACCGACC	GCACGCGCGCTTGCGCGCCACGATAGTACGCC
pFUGW-T2A-mRab23 Y79del	GAAGGCCTACCGAGGAGCCCAGGCTTGTGTGCTTGTGTTTTC	CTCCTCGGTAGGCCTTCGTGATTGCATCAAACTCCTCTTGACC
Site-directed mutagenesis using Transfer PCR		
pET28a-His-SUMO-Rab23$_{7\text{-}172}$ Y79del	TAATACGACTCACTATAGGG	CAAGGCGTACTATCGTGGCGCGCAAGCGCGCGTG

Table S2. Protein and reservoir solution conditions for crystallisation

	**hRab23$_{7-172}$-GDP**	**hRab23$_{7-172}$-GMPPNP 1**	**hRab23$_{7-172}$-GMPPNP 2**	**hRab23$_{7-172}$ Y79del-GDP**	**hRab23$_{7-172}$ Y79del-GMPPNP**
**PDB ID**	8YL3	8YIM	8YNR	8YO0	8YP0
**Space Group**	$P2_1\ 2_1\ 2_1$	$P3_1\ 2\ 1$	$P2\ 2_1\ 2_1$	$P2_1\ 2_1\ 2_1$	$P2_1\ 2_1\ 2_1$
**Protein Conditions**	15 mg/mL hRab23$_{7-172}$-GDP, 20% DMSO	20 mg/mL hRab23$_{7-172}$-GMPNP	15 mg/mL hRab23$_{7-172}$-GMPNP	15 mg/mL hRab23$_{7-172}$ Y79del-GDP	20 mg/mL hRab23$_{7-172}$ Y79del-GMPPNP
**Reservoir Solution Conditions**	0.1 M sodium acetate, pH 5.0, 0.2 M magnesium chloride and 27.5 % PEG 6000	0.1 M imidazole pH 8.0, 0.2 M sodium chloride, 0.4 M sodium phosphate monobasic / 1.6 M potassium phosphate dibasic	0.1 M SPG buffer pH 5.0 (Molecular Dimensions), 20 % PEG 1500	0.1 M SPG pH 5.0 (Molecular Dimensions), 27 % PEG 1500	0.1 M sodium acetate tribasic pH 5.0, 0.2 M sodium chloride, 25 % PEG 6000

Table S3. P values for comparisons from the GEF and GAP assays in Figure 5.

Assay	Comparison Group 1	Comparison Group 2	P Value
GEF Assay (hRab23$_{7-172}$)	Buffer	EDTA	0.7241
		Intu-Fuz	0.7241
		hRab23$_{7-172}$	0.0279
	hRab23$_{7-172}$	hRab23$_{7-172}$ & EDTA	0.1583
		hRab23$_{7-172}$ & Intu-Fuz	<0.0001 (1.4 X 10^{-10})
GEF Assay (hRab23$_{7-172}$ Y79del)	Buffer	EDTA	0.8828
		Intu-Fuz	0.8760
		hRab23$_{7-172}$ Y79del	0.0498
	hRab23$_{7-172}$ Y79del	hRab23$_{7-172}$ Y79del & EDTA	0.5174
		hRab23$_{7-172}$ Y79del & Intu-Fuz	0.8760
GAP Assay	Buffer	EVI5L	0.5693
		hRab23$_{7-172}$	<0.0001 (9.9 x 10^{-5})
		hRab23$_{7-172}$ Y79del	0.0003
	hRab23$_{7-172}$	hRab23$_{7-172}$ & EVI5L	0.0329
	hRab23$_{7-172}$ Y79del	hRab23$_{7-172}$ Y79del & EVI5L	0.6789

Table S4. P values for comparisons from cell biology assays in Figure 6.

Assay	Comparison Group 1	Comparison Group 2	P Value
Real-time qPCR (*Rab23* expression level)	Vector Control	Rab23WT	0.0002
		Rab23QL	0.0015
		Rab23SN	0.0029
		Rab23Y79del	0.0043
Real-time qPCR (*Gli1* expression level)	Vector Control	Rab23WT	0.2519
		Rab23QL	0.0387
		Rab23SN	0.0024
		Rab23Y79del	0.0091
His pull-down assay	$hRab23_{7\text{-}172}$	$hRab23_{7\text{-}172}$ Y79del	0.0018

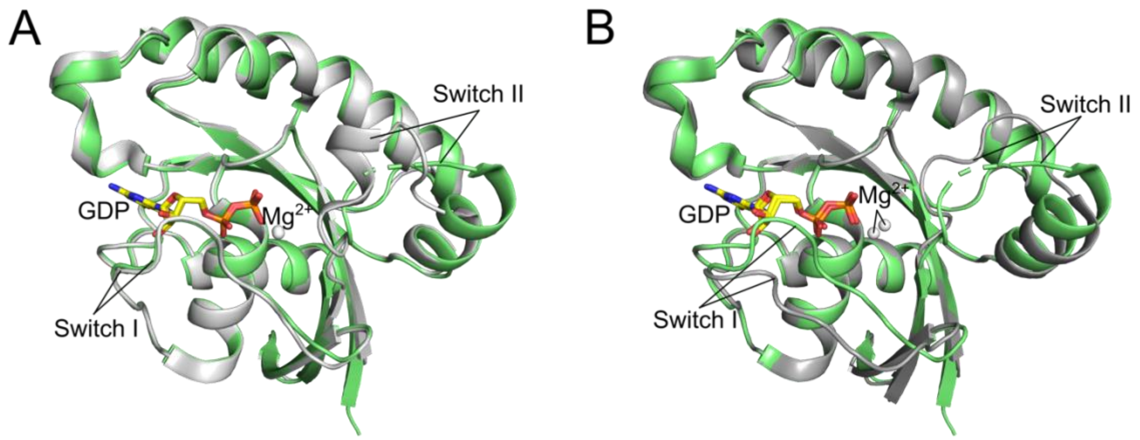

Figure S1. Structural comparison of the hRab23$_{7\text{-}172}$-GDP complex with the mRab23-GDP complex. Superimpositioning of the structure of the hRab23$_{7\text{-}172}$-GDP complex (PDB ID 8YL3) (green) with the structures of the mRab23-GDP complex (grey) in **(A)** $P2_1\ 2_1\ 2_1$ space group (PDB ID 1Z2A) (Eathiraj et al., 2005) and **(B)** $C2\ 2\ 2_1$ space group (PDB ID 1Z22) (23).

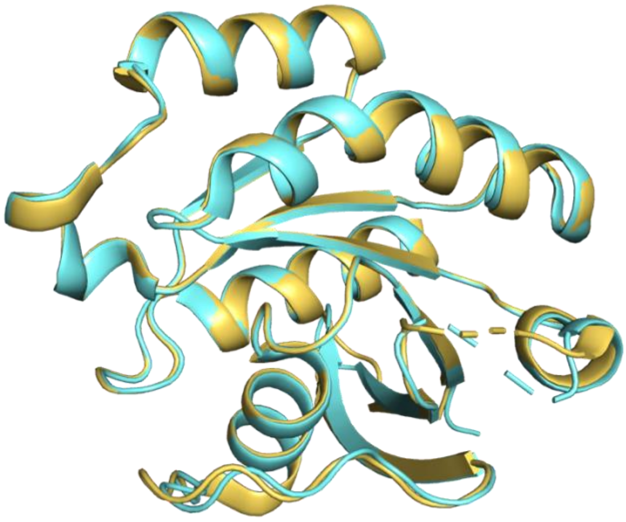

Figure S2. Structural comparison of the structures of the hRab23$_{7\text{-}172}$-GMPPNP complex in two different space groups. Superimposition of the structures of hRab23$_{7\text{-}172}$ in complex with GMPPNP in space groups *P* 3_1 2 1 (1.20 Å) (PDB ID 8YIM) (light orange) and *P* 2 2_1 2_1 (1.80 Å) (8YNR) (cyan).

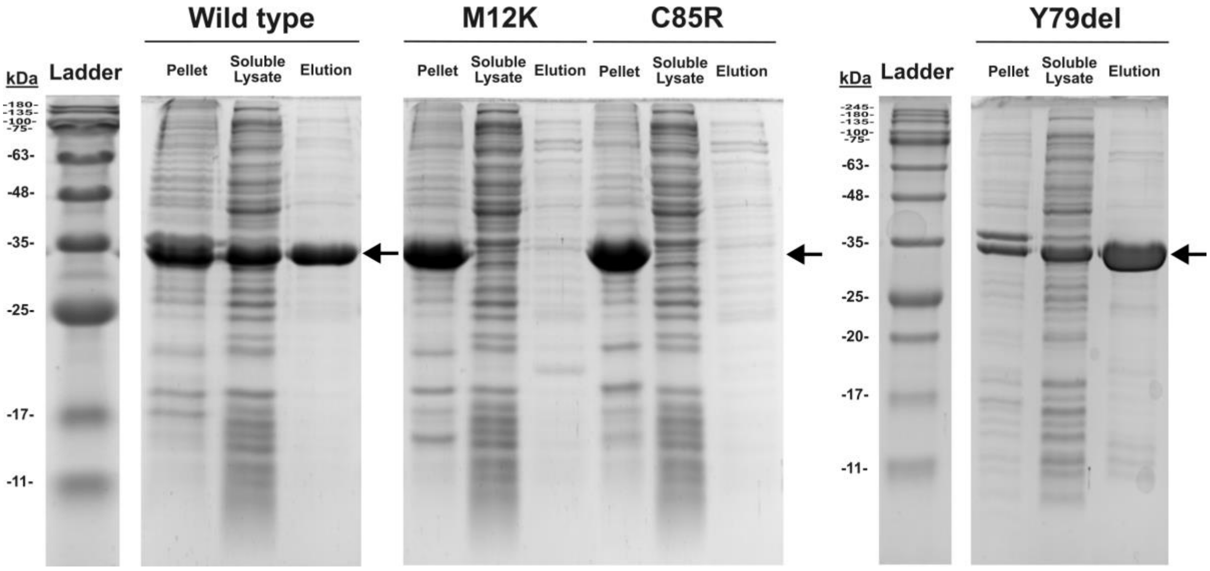

Figure S3. SDS-PAGE analysis of small scale expression and nickel affinity purification trials of hRab23$_{7\text{-}172}$ wild-type, hRab23$_{7\text{-}172}$ M12K, hRab23$_{7\text{-}172}$ C85R, and hRab23$_{7\text{-}172}$ Y79del. *E. coli* cells from 5 mL LB cultures were lysed and centrifuged to separate into insoluble (Pellet) and soluble (Soluble Lysate) fractions. The soluble lysate was then incubated with Ni-NTA agarose beads and the proteins eluted with imidazole (Elution). The fractions were analysed by SDS-PAGE.

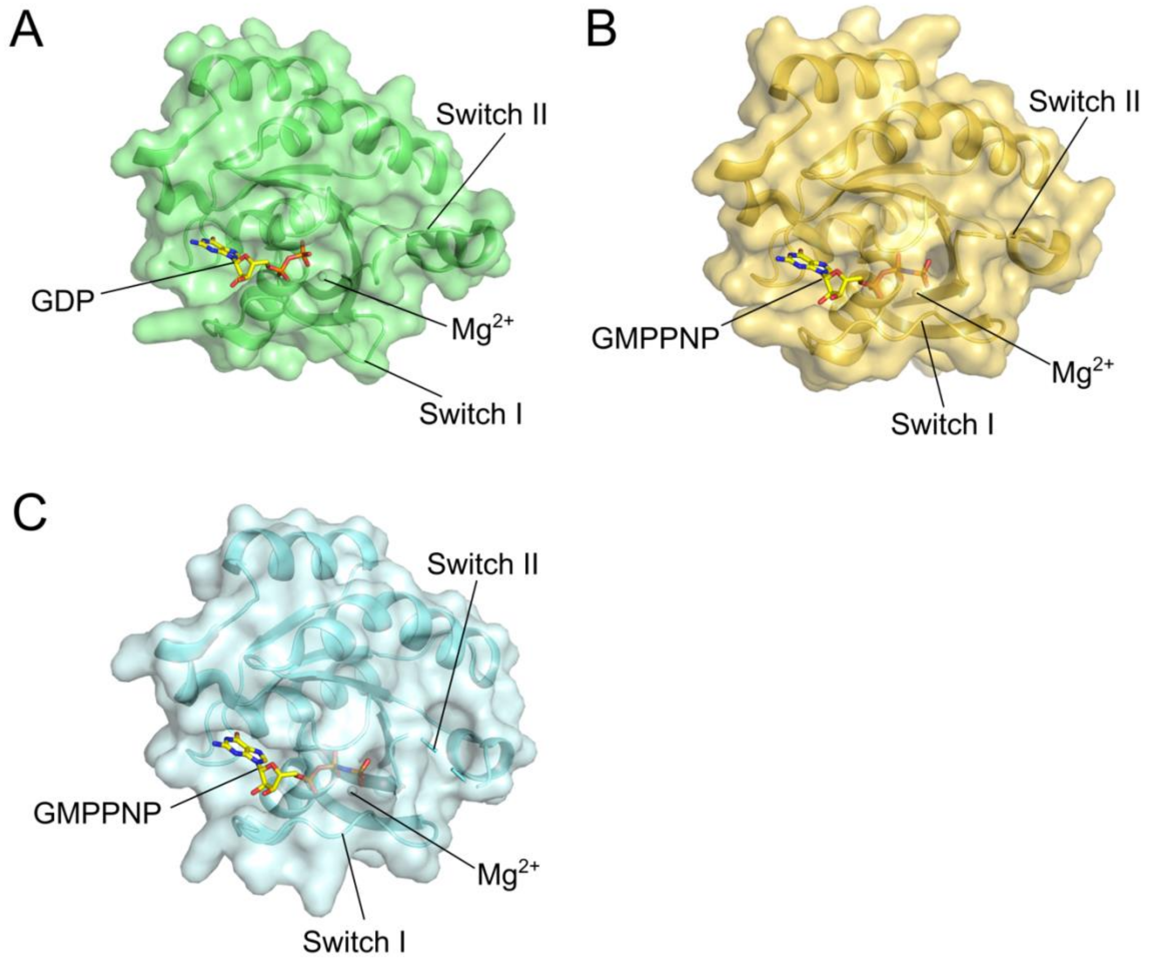

Figure S4. Surface representations of the overall structures of hRab23$_{7\text{-}172}$. (A) Structure of the hRab23$_{7\text{-}172}$-GDP complex (resolution 1.20 Å) (PDB ID 8YL3); **(B)** structure of hRab23$_{7\text{-}172}$-GMPPNP complex (resolution 1.35 Å) (PDB ID 8YIM); and **(C)** structure of hRab23$_{7\text{-}172}$-GMPPNP complex (resolution 1.80 Å) (PDB ID 8YNR). GDP/GMPPNP are shown in sticks (C, yellow; O, red; N, blue; P, orange); Mg^{2+} in white spheres.

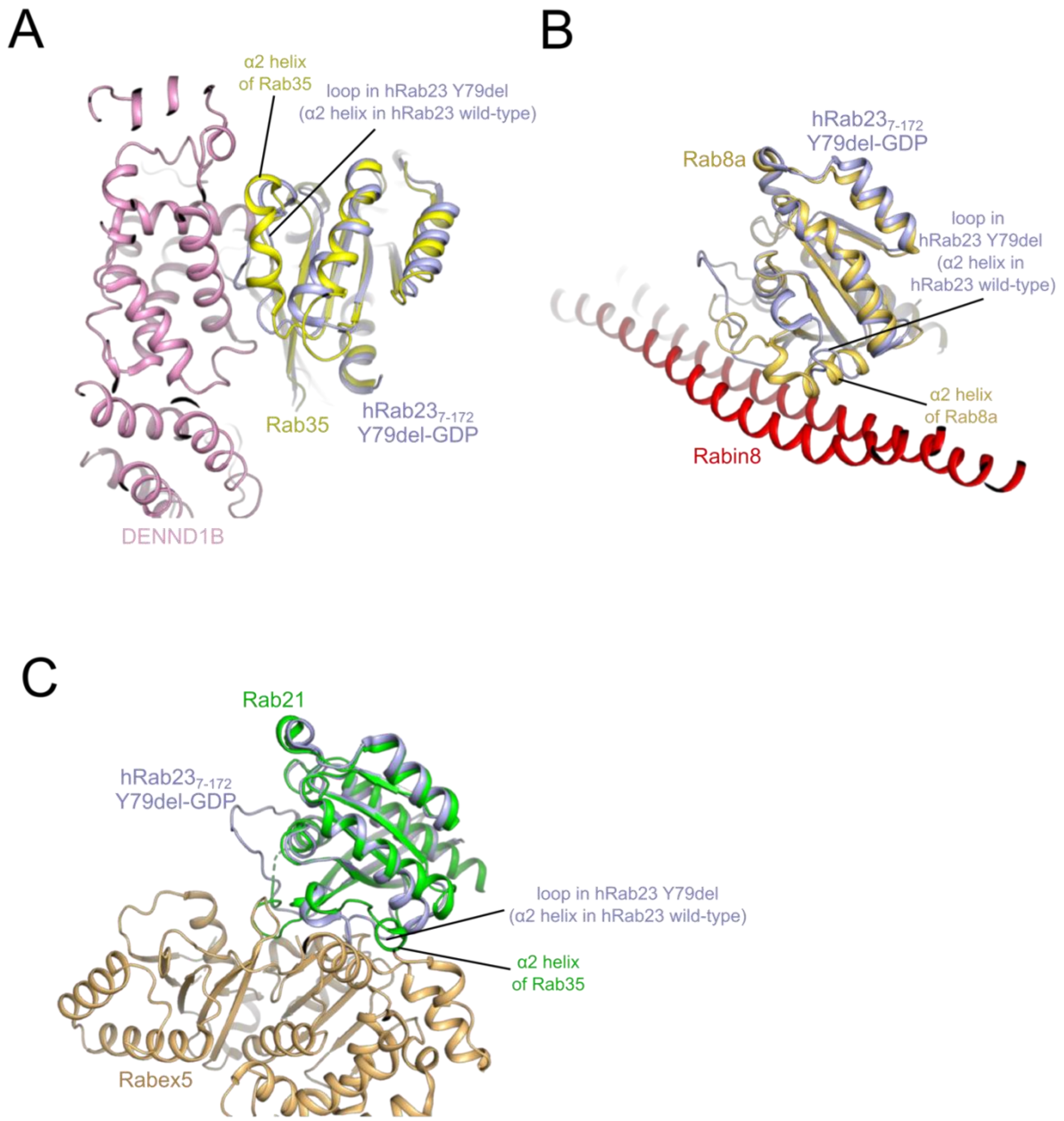

Figure S5. Interactions between representative Rab proteins and their respective GEFs. Superimposition of the structure of the hRab23$_{7\text{-}172}$ Y79del-GDP complex (PDB ID 8YO0) (light blue) onto structures of **(A)** Rab35 (yellow) in complex with DENND1B (pink) (PDB ID 3TW8) (30), **(B)** Rab8a (yellow orange) in complex with Rabin8 (red) (PDB ID 4LHX) (31), and **(C)** Rab21 (green) in complex with Rabex5 (light orange) complex (PDB ID 2OT3) (29). Note that the α2 helices of the Rab proteins interact with their respective GEFs while the corresponding region in the hRab23$_{7\text{-}172}$ Y79del protein forms a loop as a result of the mutation, potentially disrupting the interactions between hRab23 Y79del with its GEF.

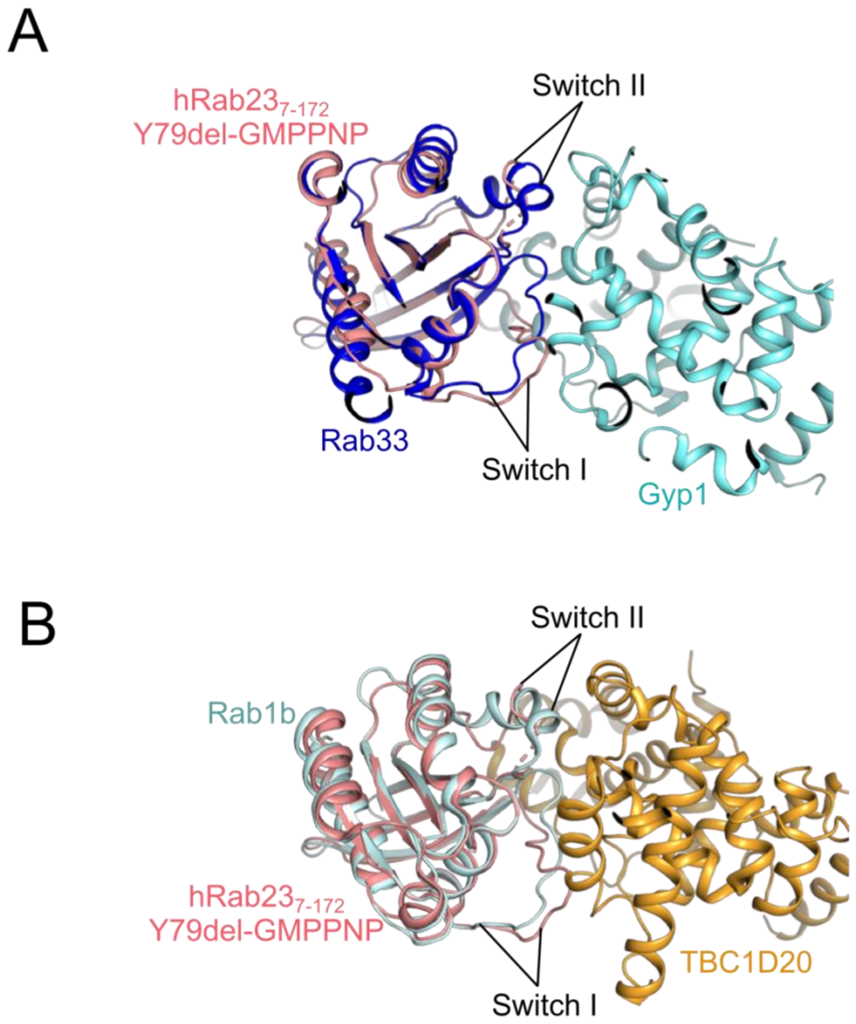

Figure S6. Interactions between representative Rab proteins and their respective GAPs. Superimposition of the structure of the hRab23$_{7\text{-}172}$ Y79del-GMPPNP complex (PDB ID 8YP0) (salmon) onto the structures of **(A)** Rab33 (dark blue) in complex with Gyp1 TBC domain (cyan) (PDB ID 2G77) (32), and **(B)** Rab1b (light cyan) in complex with TBC1D20 (orange) (PDB ID 4HLQ) (33). Note that Switches I and II of the Rab proteins interact with their respective GAPs while the corresponding regions in the hRab23$_{7\text{-}172}$ Y79del protein are in different conformations as a result of the mutation, potentially disrupting the interactions between Rab23 Y79del with its GAP.

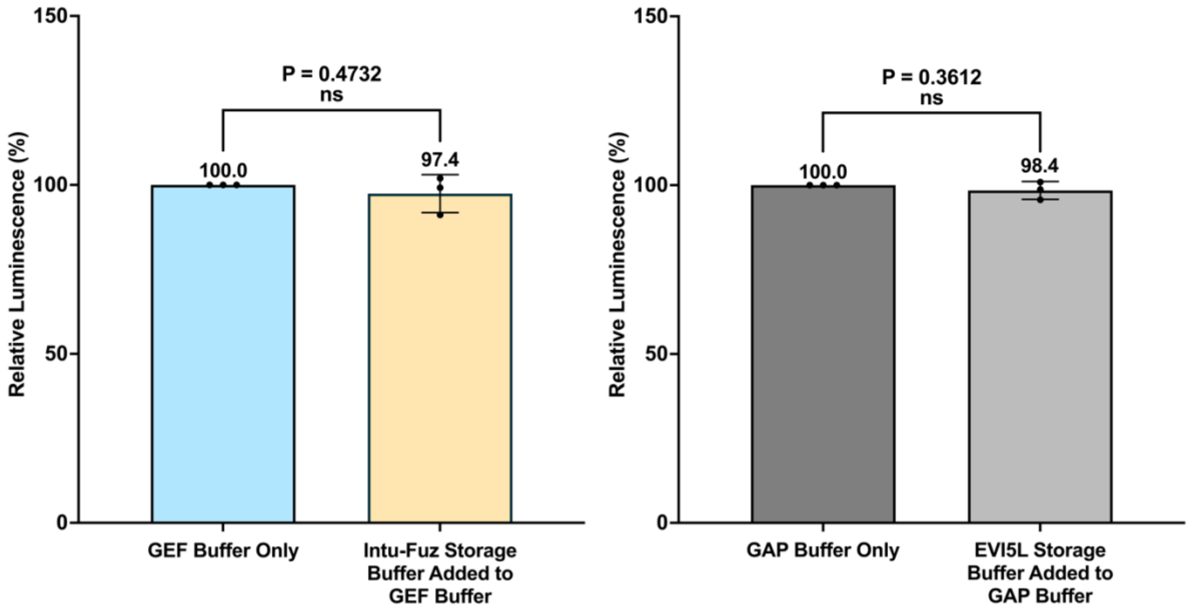

Figure S7. *In vitro* GTPase-Glo^{TM} assay of Intu-Fuz and EVI5L storage buffers. Corresponding volumes of protein storage buffers as would be for assays involving addition of Intu-Fuz and EVI5L proteins were added to the GEF and GAP buffers to determine their effects on luminescence. Note that both the Intu-Fuz and EVI5L buffers did not significantly result in relative luminescence change relative to buffer only controls. Heights of the bar graphs indicate the mean (n = 3; assay triplicates); black dots indicate individual data points; error bars indicate standard deviation of the mean (n = 3; assay triplicates). ns = not significant (unpaired 2-tailed Student's t-test).
